# Loss of progesterone receptor membrane component 1 promotes hepatic steatosis via the induced *de novo* lipogenesis

**DOI:** 10.1038/s41598-018-34148-6

**Published:** 2018-10-24

**Authors:** Sang R. Lee, Sun Woo Kwon, Pelin Kaya, Young Ho Lee, Jong Geol Lee, Globinna Kim, Geun-Shik Lee, In-Jeoung Baek, Eui-Ju Hong

**Affiliations:** 1 College of Veterinary Medicine, Chungnam National University, Daejeon, 34134 Republic of Korea; 2 Department of Convergence Medicine, University of Ulsan College of Medicine, Asan Medical Center, Seoul, 05505 Republic of Korea; 3 College of Veterinary Medicine and Institute of Veterinary Science, Kangwon National University, Chuncheon, 24341 Republic of Korea

## Abstract

Non-alcoholic fatty liver disease (NAFLD) results from triglyceride accumulation within the liver and some of them advances to non-alcoholic steatohepatitis (NASH). It is important to note that in NAFLD development, hepatic *de novo* lipogenesis (DNL) derives from excess carbohydrates and fats under a condition of excess energy through β-oxidation. As a main regulator for DNL, sterol regulatory element-binding protein 1 (*Srebp-1*) forms complex with progesterone receptor membrane component 1 (*Pgrmc1*). To investigate whether *Pgrmc1* may have a notable effect on DNL *via* SREBP-1 activation, we generated *Pgrmc1* knockout (KO) mice and fed a high fat diet for one month. High-fat-fed *Pgrmc1* KO mice showed a substantial increase in levels of hepatic TG accumulation, and they were predisposed to NAFLD when compared to WT mice. Loss of *Pgrmc1* increased mature SREBP-1 protein level, suggesting that induction of hepatic steatosis in *Pgrmc1* KO mice might be triggered by *de novo* lipogenesis. Moreover, *Pgrmc1* KO mice were also more vulnerable to early stage of NASH, showing high levels of alanine aminotransferase, obesity-linked pro-inflammatory cytokines, and fibrosis markers. This is interesting because *Pgrmc1* involves with the first step in regulating the hepatic de novo lipogenesis under an excess energy condition.

## Introduction

Non-alcoholic fatty liver disease (NAFLD) is a recently emerging problem mostly prevalent in western societies where obesity rates are high due to consumption of a high fat diet^1^. NAFLD typically results from hepatic triglyceride accumulation and accompanies steatosis and hepatitis^2^. When NAFLD becomes intensified, a small portion of NAFLD (30%) progresses to non-alcoholic steatohepatitis (NASH). Although less than half of NAFLD progresses to NASH, the progression can lead to serious liver injury such as cirrhosis or hepatocellular carcinoma (HCC). As mechanisms underlying the progression of NAFLD to NASH are not well characterized^3–6^, it is important to find a way to prevent NAFLD to reduce the risk of NAFLD progression to NASH.

Hepatic *de novo* lipogenesis (DNL) is the metabolic pathway that enables accumulation of fatty acids in liver under condition of a high-carbohydrate and a high-fat diet^7^. It is also known that DNL levels are determined by the sterol regulatory element binding protein (*Srebp*). *Srebp* normally exists in the endoplasmic reticulum (ER) of the liver and consists of two members, *Srebp-1* and *Srebp-2*. *Srebp-1* regulates fatty acid metabolism and *Srebp-2* regulates cholesterol synthesis by involving the pathway between mevalonate and lanosterol^8,9^. *Srebp-1* is also one of the most significant transcriptional regulator for DNL in the liver, along with Lxr and Ppar, and they interact with each other in the DNL pathway^10–14^. SREBP-1 generally exists as an inactivated form and binds with Srebp cleavage-activated protein (SCAP), which forms complex with insulin induced gene 1 (INSIG-1) and progesterone receptor membrane component 1 (PGRMC1) in the ER of the liver^15^. After SCAP is activated by extracellular signaling, it dissociates with INSIG-1 protein and activates the SREBP-1^16^. Then, SREBP-1 is transferred to the golgi complex and acts as the gene regulator for fatty acid synthesis and triglyceride synthesis^17^. Interestingly, PGRMC1 also plays a role in SREBP-1 activation^18^. As a novel surface cell receptor, PGRMC1 protein has been detected in various tissues such as the liver, lung, kidney, and brain^19–21^. Furthermore, PGRMC1 is a member of the PGRMC1/INSIG-1 complex, which is located in the ER of human and rodent liver. In addition to its role as a regulator of cancer cell survival, *Pgrmc1* also affects cholesterol synthesis and steroidogenesis^18,22–24^. Moreover, a recent study revealed that PGRMC1 binds to INSIG-1 and subsequently regulates SCAP and SREBP-1 that are closely related to triglyceride synthesis^25^.

In the present study, we focused on the role of *Pgrmc1* as a regulator of *Srebp-1* in the hepatic DNL pathway. In our preliminary data, we have evidence that Pgrmc1 and *Srebp-1* are associated by inverse-proportional expression. To investigate whether *Pgrmc1* actually regulates lipogenesis, we generated *Pgrmc1* knockout (KO) mice, fed them with a high fat diet, and assessed whether the loss of *Pgrmc1* predisposes mice to NAFLD and results in the buildup of fatty acids via the DNL pathway.

## Result

### Generation of *Pgrmc1* knockout mice

**Figure 1:**
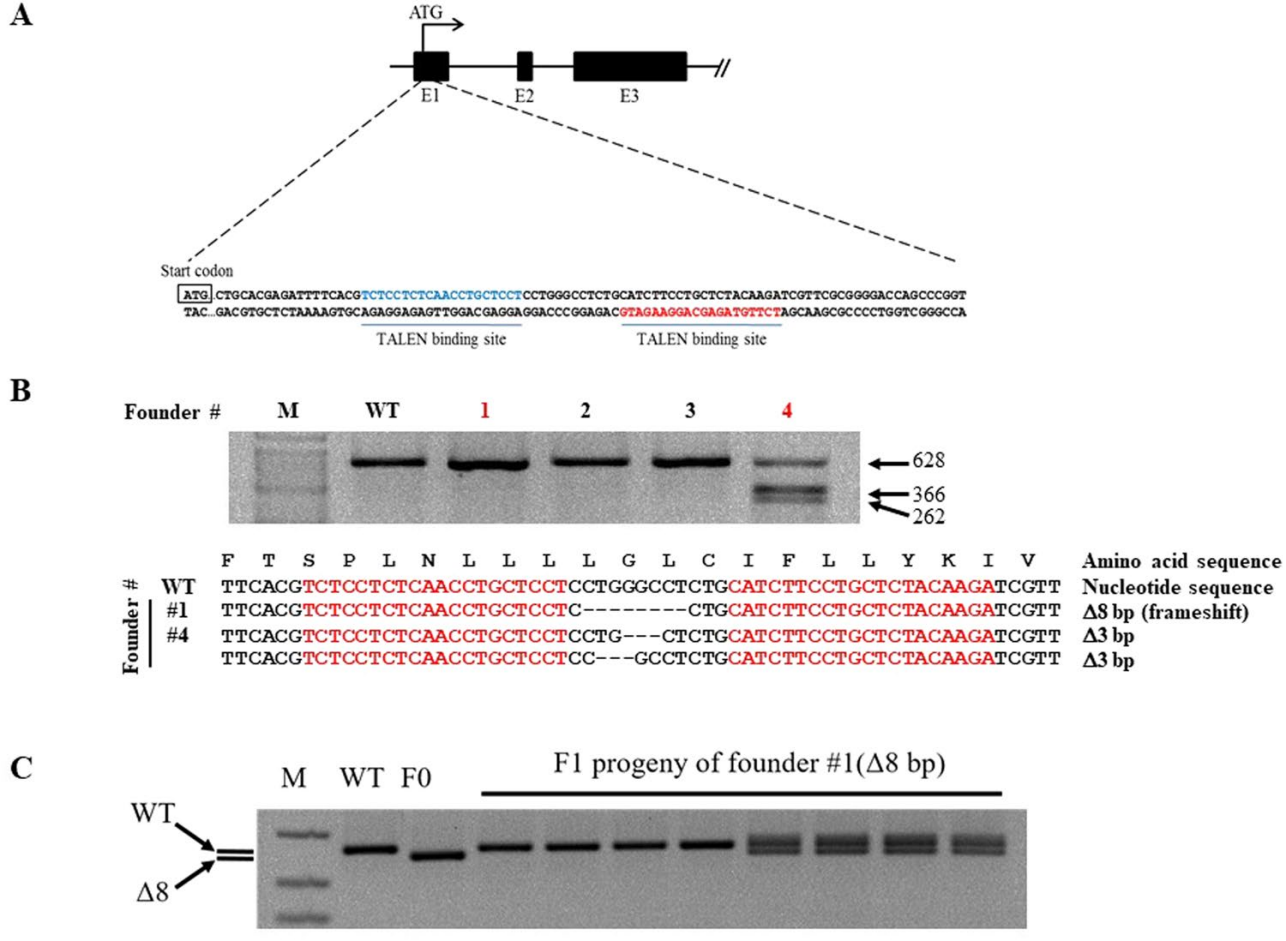
Gene targeting and generation of TALEN-mediated *Pgrmc1* mutant mice. (**A**) Target region of Pgrmc1-TALEN in mouse *Pgrmc1 locus*. (**B**) T7E1-based mutation detection assays and sequencing analysis. The numbers indicated wild type (WT, in black) and mutant (in red) mice. Colored sequences of *Pgrmc1* indicate TALEN binding sites. “-” denoted deleted nucleotides. M, a molecular size marker. (**C**) *Pgrmc1* mutant founder #1 was crossed to WT mouse and the genotypes of the pups were determined by PCR analysis.

**Figure 2:**
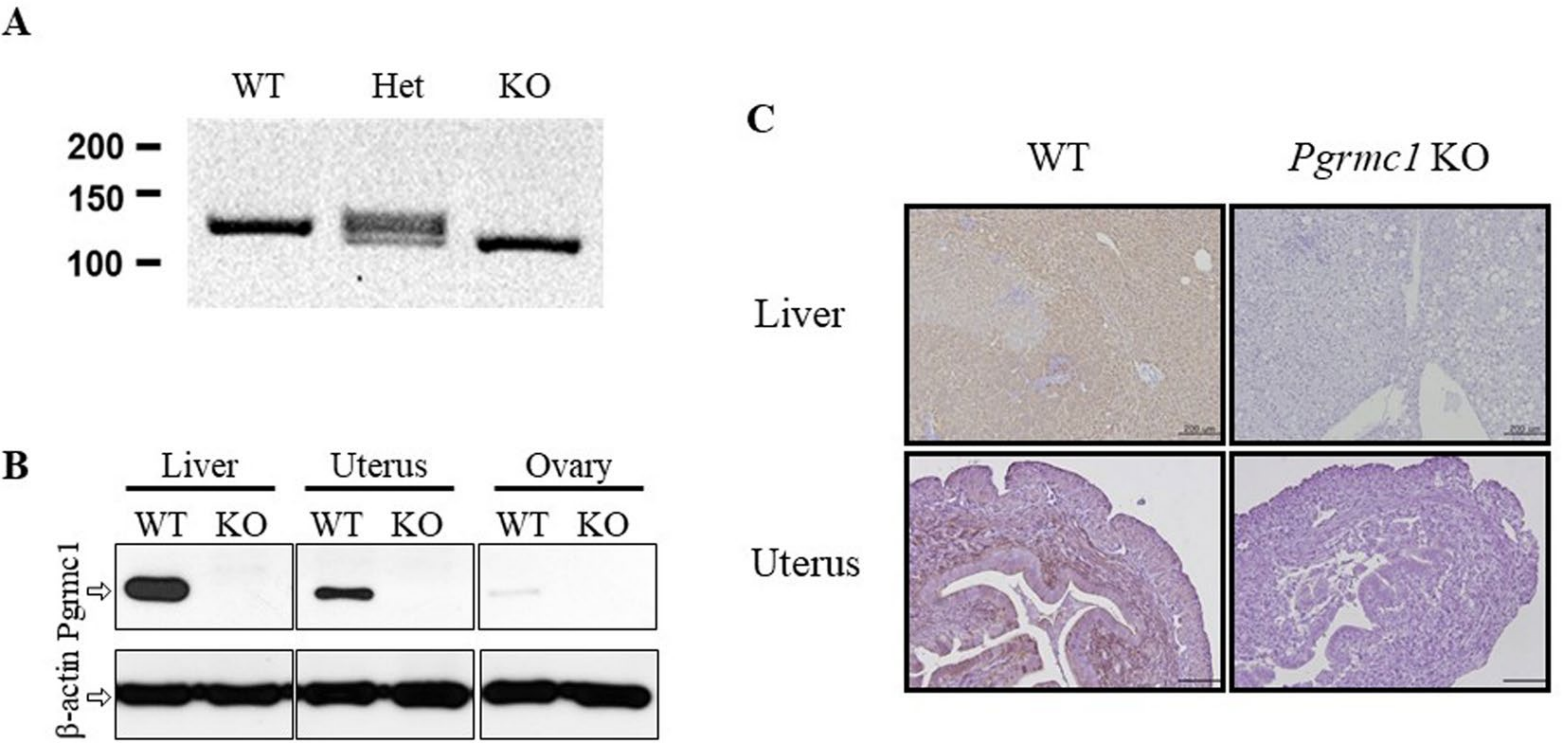
No detectable PGRMC1 protein in *Pgrmc1* knockout mice. (**A**) Genomic DNA showing PCR amplification of wild-type (WT), *Pgrmc1* heterozygote (Het) and homozygote (KO) alleles. *Pgrmc1* WT band is shown at 128 bp, whereas mutant band is at 120 bp. (**B**) Western blot showing ablation of the PGRMC1 protein. (**C**) Immunohistochemistry from PGRMC1 WT and Pgrmc1 knockout mice. (Liver; 8-week old male, uterus; ovariectomized and P4 injected mice. Scale bar, 200 µm for liver and 100 µm for uterus).

Considering that *Pgrmc1* is highly expressed in liver, it can be speculated that *Pgrmc1* is related to general liver function. To investigate role of *Pgrmc1*, we generated the knockout mice using transcription activator-like effector nuclease (TALEN). An illustration of genome targeting and editing strategy in exon 1 was as shown in Fig. 1A. Whether Pgrmc1 allele was ablated was investigated using T7 endonuclease (T7E1) assay and genomic DNA sequencing was performed to elucidate 8 base pair (bp) deletion with a frameshift in founder #1 (Fig. 1B). By crossing with wild-type, the progeny of founder #1 showed wild-type and heterozygote band exclusively, indicating that germline transmission was successful (Fig. 1C). Even though TALEN has successfully deleted on-target sites at exon 1, potential off-target sites of *Pgrmc1*-TALEN were analyzed. Sequences of expected off-target sites are as listed in Table 1. With 3–5 base pairs of mismatch, chromosomes 1, 5, 6, 12, and 17 were examined by T7E1 assay and genotyping were conducted. Compared with wild-type band, none of the chromosomes were off-targeted (Fig. S1). The absence of Pgrmc1 was confirmed by PCR genotyping, western blot analysis, and immunohistochemistry in liver (Fig. 2A–C). Wild-type allele was detected as 128 bp and mutant allele was 120 bp in size (Fig. 2A). PGRMC1 protein was detected in protein extracts from the tissues of wild-type, while this band was absent from those of *Pgrmc1* KO mice, which confirms the ablation of the PGRMC1 protein (Fig. 2B). Immunohistochemistry of the liver and uterus also demonstrated that PGRMC1 protein was completely depleted in *Pgrmc1* KO mice (Fig. 2C). To sum it up, expression tests confirmed that functional PGRMC1 was completely depleted in knockout mice.

**Table 1:** Potential off-target sites of Pgrmc1-TALEN.

Locus	Left TALEN binding site	Mismatch #	Right TALEN binding site	Mismatch #
***Pgrmc1***	TCTCCTCTCAACCTGCTCCT		TCTTGTAGAGCAGGAAGATG	
*Chr1:170565021–170565072*	TCTCCTCTtcACCTcCTCCc	4	TCTTGaAAAAatAAAAAAcA	4
*Chr17:85818163–85818214*	TCTCtTCTCAcCCTcCTCCc	4	TCcTGTccAGatGGAAAATG	5
*Chr12:60494489–60494540*	TacCCTCTCcACCTACTCCT	3	TtTgGTGGAcaAGGAAAATG	4
*Chr6:60182750–60182801*	TCTCCTaTCcACCcACTCCc	4	TCcaGTAcAGgAAAAAGccA	6
*Chr5:138161764–138161815*	TCcCCTCTCAcaCTGCTCCT	3	TaTcATtGAcaAAGAAtATt	7

**Table 2:** Primers used for real-time or conventional PCR.

Gene name	Upper primer (5′-3′)	Lower primer (5′-3′)	Species
*Rplp0*	GCA GCA GAT CCG CAT GTC GCT CCG	GAG CTG GCA CAG TGA CCT CAC ACG G	Mouse
*Lxr*	CTC AAT GCC TGA TGT TTC TCC T	TCC AAC CCT ATC CCT AAA GCA A	Mouse
*Pparγ*	GAC CAC TCG CAT TCC TTT GAC	ATG CAG GTT CTA CTT TGA TCG C	Mouse
*Srebp1*	GCC CGG ACA CAC CAG CTC	TGC CCA GGA GCC GAC AGG	Mouse
*Scd1*	CTG TTC GTT AGC ACC TTC TTG	CAG AGT AGT CGA AGG GGA AG	Mouse
*Tnf*	CCT GTA GCC CAC GTC GTA G	GGG AGT AGA CAA GGT ACA ACC C	Mouse
*Il-6*	CTG CAA GAG ACT TCC ATC CAG	AGT GGT ATA GAC AGG TCT GTT GG	Mouse
*Il-1β*	GAA ATG CCA CCT TTT GAC AGT G	CTG GAT GCT CTC ATC AGG ACA	Mouse
*Acc*	TGT GGG CTG GCT GGG GTC	CTG CCA CTC CAG GGA AGA G	Mouse
*Acly*	CAT CGG CGT TGC GTT TGT GG	GCC CAT ACT CCT TCC TAG CA	Mouse
*Agpat1*	TAA GAT GGC CTT CTA CAA CGG C	CCA TAC AGG TAT TTG ACG TGG AG	Mouse
*Mogat1*	TGG TGC CAG TTT GGT TCC AG	TGC TCT GAG GTC GGG TTC A	Mouse
*Tgfb*	GAC GTC ACT GGA GTT GTA CG	GGT TCA TGT CAT GGA TGG TG	Mouse
*Insig-1*	CAC GAC CAC GTC TGG AAC TAT	TGA GAA GAG CAC TAG GCT CCG	Mouse
*Scap*	TGG AGC TTT TGA GAC TCA GGA	TCG ATT AAG CAG GTG AGG TCG	Mouse
*Cd36*	ATG GGC TGT GAT CGG AAC TG	GTC TTC CCA ATA AGC ATG TCT CC	Mouse
*Pparα*	GCG TTT CCT GAG ACC CTC G	GGC TCT CTG TGT CCA CCA TG	Mouse
*Mcad*	AGG TTT CAA GAT CGC AAT GG	CTC CTT GGT GCT CCA CTA GC	Mouse
*Cpt2*	CAG CAC AGC ATC GTA CCC A	TCC CAA TGC CGT TCT CAA AAT	Mouse
*Acox1*	TTG GAA ACC ACT GCC ACA TA	AGG CAT GTA ACC CGT AGC AC	Mouse
*Hsl*	TCC CTC AGT ATC TAG GCC AGA	GGC TCA TTT GGG AGA CTT TGT TT	Mouse
*Apo B*	TTG GCA AAC TGC ATA GCA TCC	TCA AAT TGG GAC TCT CCT TTA GC	Mouse
*Apo E*	CTG ACA GGA TGC CTA GCC G	CGC AGG TAA TCC CAG AAG C	Mouse
*Ldlr*	AGT GGC CCC GAA TCA TTG AC	CTA ACT AAA CAC CAG ACA GAG GC	Mouse
*Vldlr*	GGC AGC AGG CAA TCG AAT G	GGG CTC GTC ACT CCA GTC T	Mouse
*Pgrmc1*	GGC AAG GTG TTC GAC GTG A	GTC CAG GCA AAA TGT GGC AA	Mouse
*Fabp4*	CCG CAG ACG ACA GGA AGG T	AGG GCC CCG CCA TCT	Mouse
*Fatp2*	GCT GAC ATC GTG GGA CTG GT	TTC GAC CCT CAT GAC CTG GC	Mouse
*Fatp5*	ACC ACT GGA CTC CCA AAG CC	AGG ACA GCA CGT TGC TCA CT	Mouse
*Fabp5*	TCT TAA GGA TCT CGA AGG GAA	CTT CCT AAG AGC CAG TCC TAC T	Mouse
*PGRMC1*	AAA GGC CGC AAA TTC TAC GG	CCC AGT CAC TCA GAG TCT CCT	Human
*SREBP1*	CGG AAC CAT CTT GGC AAC AGT	CGC TTC TCA ATG GCG TTG T	Human

### Hepatic TG accumulation significantly increases in *Pgrmc1* KO mice

**Figure 3:**
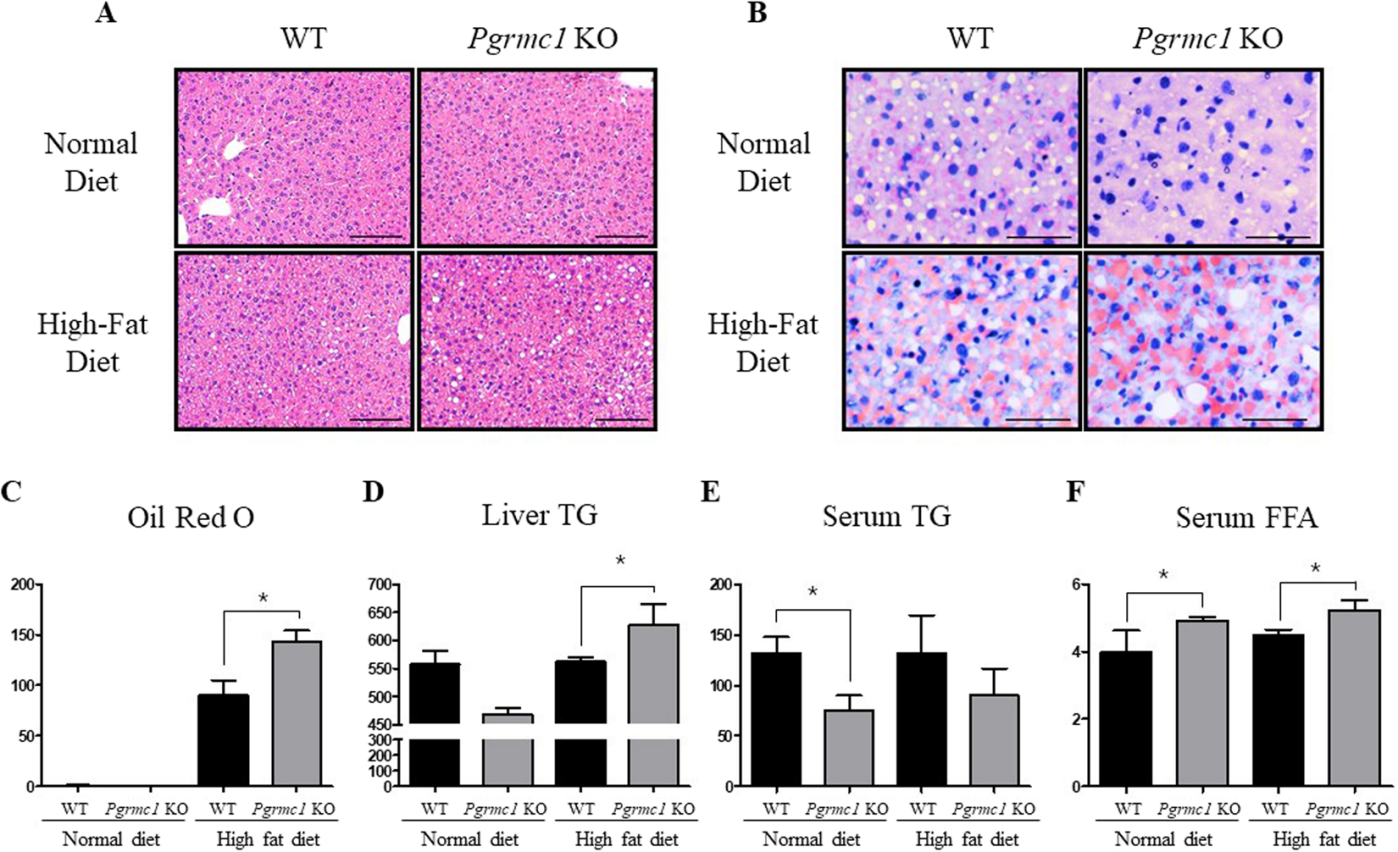
Loss of *Pgrmc1* increases hepatic TG accumulation. Normal diet and high fat diet were fed for 1 month to WT and *Pgrmc1* KO mice. (**A**) Lipid droplets are characterized in H&E staining (Scale bar, 50 µm). (**B**) Oil Red O staining presents TG accumulation in hepatocytes (Scale bar, 25 µm). (**C**) Quantification of Oil Red O was analyzed by Image J as setting red cells for positive standard. (**D**) Total lipid from liver was extracted by folch method and level of TG was measured by colorimetric method in 550 nm. (**E**) Level of TG (triglycerides) in plasma showed inverse proportional level compared to liver TG. (**F**) Level of FFA in plasma was increased in *Pgrmc1* KO mice. Values represent means ± SD. *P < 0.05. Number of mice used in experiment is at least 3 for each group.

Control WT and *Pgrmc1* KO groups were fed a normal diet and experimental WT and *Pgrmc1* KO groups were fed a high fat diet for 1 month. After necropsy, high-fat-fed *Pgrmc1* KO mice showed increased lipid accumulation within the liver when compared to high-fat-fed WT mice. Lipid droplets observed in H&E stained liver sections were remarkably larger in high-fat-fed *Pgrmc1* KO mice when compared to that of WT mice (Fig. 3A). In addition, on Oil-Red-O staining, lipid droplets were stained with red orange color and its quantification showed an increase (*P* < 0.05, 1.43 fold) in high-fat-fed *Pgrmc1* KO mice (Fig. 3B,C). Similarly, after extracting lipids from the liver, triglyceride levels were measured with a commercial kit mentioned above and it showed significantly increased (*P* < 0.05, 1.12 fold) in the liver of high-fat-fed *Pgrmc1* KO mice when compared to high-fat-fed WT mice (Fig. 3D). These results suggest that numerous lipid droplets in Oil-Red-O staining were triglycerides, which accumulated within the liver of high-fat-fed *Pgrmc1* KO mice. On the other hand, there was a slight decrease (*P* = 0.14, 68.5%) in serum TG levels in high-fat-fed *Pgrmc1* KO mice when compared to high-fat-fed WT mice and *Pgrmc1* KO mice fed a normal diet also showed a decrease (*P* < 0.05, 56.8%) in serum TG levels when compared to WT mice fed a normal diet (Fig. 3E). Meanwhile, serum free fatty acid (FFA) levels in high-fat fed *Pgrmc1* KO mice significantly increased (*P* < 0.05, 1.16 fold) than those of high-fat-fed WT mice (Fig. 3F). Similarly, *Pgrmc1* KO mice fed a normal diet showed significantly increased (*P* < 0.05, 1.18 fold) serum FFA levels when compared to WT mice fed a normal diet (Fig. 3F).

### TG accumulation in *Pgrmc1* KO liver links to increase of *Srebp-1* level

**Figure 4:**
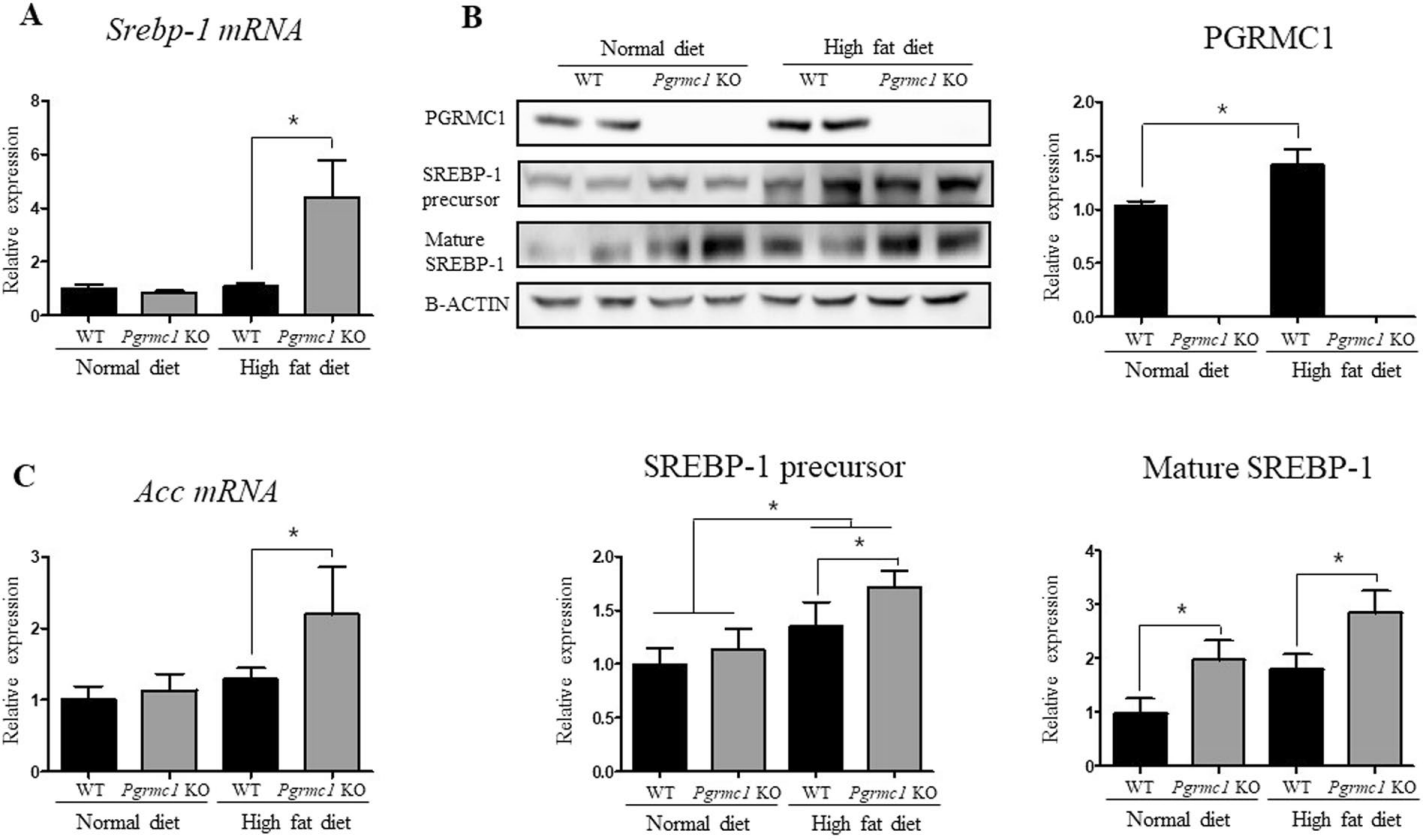
Loss of *Pgrmc1* increases level of *Srebp-1*. (**A**) Substantially increased mRNA level of *Srebp-1* in liver of high-fat-fed *Pgrmc1* KO mice. (**B**) Protein level of PGRMC1 was increased after high fat diet. Increased protein level of SREBP-1 precursor in livers of high-fat-fed group, and comparatively more increased protein level of mature SREBP-1 in liver of high-fat-fed *Pgrmc1* KO mice. (**C**) Increased mRNA level of *Acc* in liver of high-fat-fed *Pgrmc1* KO mice. *Rplp0* mRNA was used as an internal control in real time PCR. Beta actin was used as an internal control in western blot. Values represent means ± SD of at least 3 experiments. *P < 0.05. Number of mice used in experiment is at least 3 for each group.

First, we assessed transcriptional regulator genes involved in fatty acid synthesis, such as *Pparγ*, *Lxr*, and *Srebp-1*. As a result, *Srebp-1* mRNA level significantly increased (*P* < 0.05, 4.02 fold) in high-fat-fed *Pgrmc1* KO mice when compared to high-fat-fed WT mice (Fig. 4A). Although *Pparγ* and *Lxr* mRNA levels increased in high fat diet, the differences between WT mice and *Pgrmc1* KO mice were not observed (Fig. S2A). Meanwhile, protein levels of SREBP-1 precursor were increased in WT and *Pgrmc1* KO mice fed a high fat diet (*P* < 0.05, 3.1 fold and 2.8 fold) than normal diet, but the differences between WT mice and *Pgrmc1* KO mice were not observed (Fig. 4B). Rather, protein levels of mature SREBP-1 were significantly increased in *Pgrmc1* KO mice fed a normal diet (2.01 fold) and high fat diet (1.53 fold), when compared to those of WT mice (P < 0.05, Fig. 4B). These results suggest that level of active SREBP-1 protein is induced by loss of PGRMC1. Accordingly, among the genes directly controlled by SREBP-1, *Acc* mRNA levels was increased (*P* < 0.05, 1.7 fold) in high-fat-fed *Pgrmc1* KO mice when compared to high-fat fed WT mice (Fig. 4C). In addition, fatty acid esterification enzymes, *Agpat1* (1.94 fold) and *Mogat1* (2.99 fold), were significantly (*P* < 0.05) increased in mRNA levels of high-fat-fed *Pgrmc1* KO mice than high-fat-fed WT mice (Fig. S2B). These results suggest that TG accumulation was promoted under condition of increase of *Srebp-1* level, as a phenotype of *Pgrmc1* KO mice.

Interestingly, both mRNA (2.09 folds, Fig. S3) and protein (1.43 fold, Fig. 4B) level of *Pgrmc1* were increased (*P* < 0.05) in high-fat-fed WT mice than WT mice fed a normal diet, suggesting that *Pgrmc1* can also be used as potential biomarker in steatosis. Meanwhile, *Insig-1*, which forms complex with *Pgrmc1*, also significantly showed increase (*P* < 0.05, 3.74 and 2.11 fold) in mRNA level in *Pgrmc1* KO mice, when compared to WT mice in normal diet or high fat diet (Fig. S3). Likewise, *Scap*, which forms with *Pgrmc1* and *Insig-1*, increased in mRNA level (1.29 fold, *P* = 0.33) in *Pgrmc1* KO mice, but showed no significance (Fig. S3).

### Loss of *Pgrmc1* promotes steatosis-induced NASH

**Figure 5:**
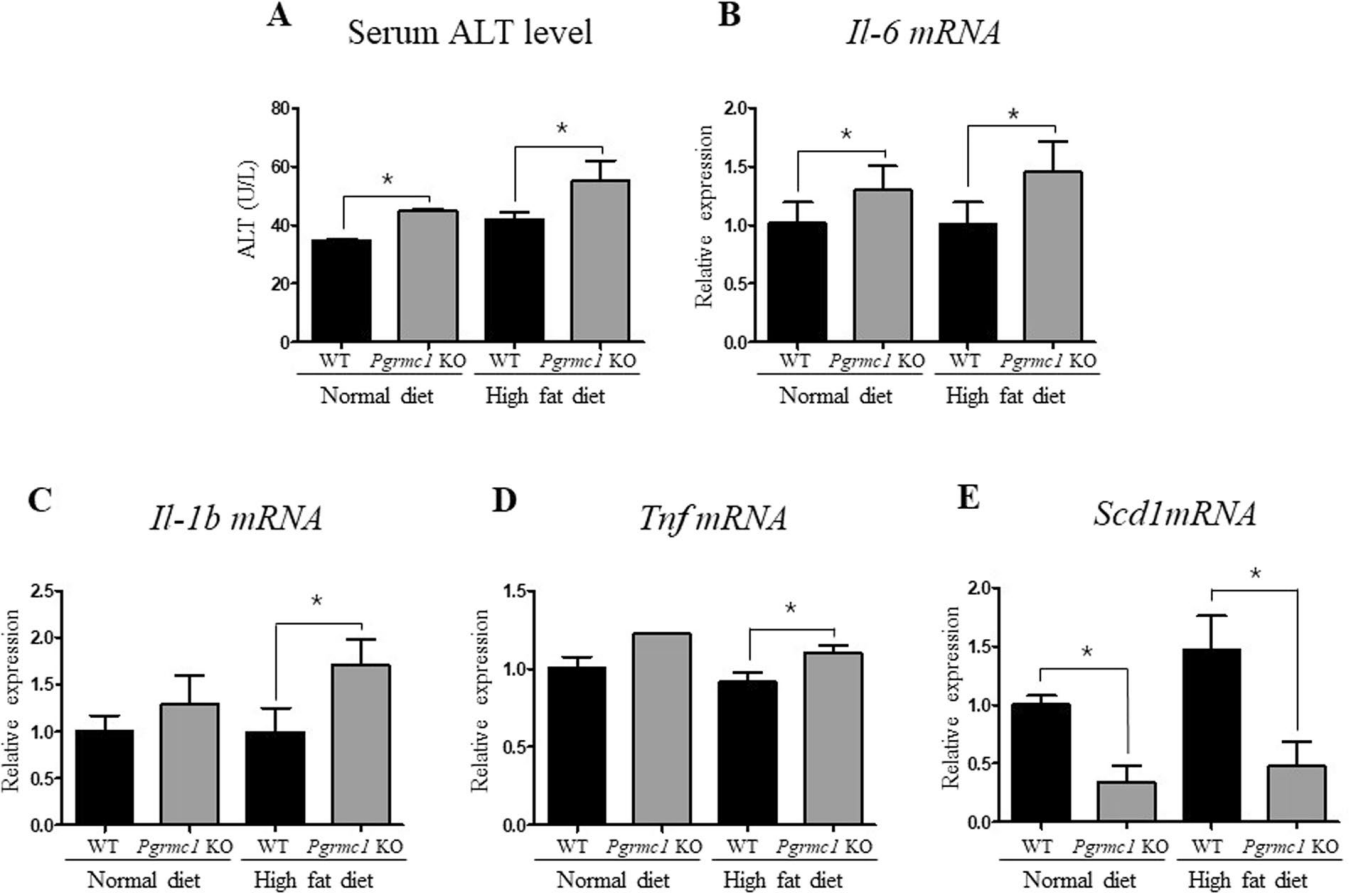
*Pgrmc1* KO mice possesses more susceptible condition for NASH with inflammation. (**A**) ALT levels in plasma indicates hepatic injury relates to inflammation. (**B**–**D**) Increased mRNA level of pro-inflammatory cytokines in high-fat-fed *Pgrmc1* KO mice. (E) Decreased mRNA level of Scd1 gene in high-fat-fed *Pgrmc1* KO mice. *Rplp0* mRNA was used as an internal control in real time PCR. Values represent means ± SD of at least 3 experiments. *P < 0.05. Number of mice used in experiment is at least 3 for each group.

As a hepatic injury marker, ALT levels were measured in serum. As a result, normal diet (1.29 fold) and high-fat-fed (1.26 fold) *Pgrmc1* KO mice showed significantly (*P* < 0.05) higher levels of ALT than those of WT mice fed with a normal diet and high fat diet (Fig. 5A). Moreover, to assess the level of genes followed by hepatic inflammation, we analyzed *Tnf* (1.2 fold), *Il-6* (1.32 fold), and *Il-1β* (1.72 fold) mRNA levels and observed significant (*P* < 0.05) increases in high-fat-fed *Pgrmc1* KO mice than those of high-fat-fed WT mice (Fig. 5B–D). In addition, we monitored macrophage infiltration in liver using a F4/80 antibody, macrophage marker, F4/80. Although liver has resident macrophage like Kupffer cells, the increased macrophage (F4/80-positive) infiltration in *Pgrmc1* KO mice suggests possibility for inflammation of NASH (Fig. S4). This result also suggests that the macrophage infiltrate the liver in response to NAFLD challenge. Likewise, the mRNA level of *Scd1*, which is protective to lipotoxicity, showed significant decrease (*P* < 0.05) in *Pgrmc1* KO mice fed a normal diet (33.8%) and high fat diet (32.5%) when compared to those of WT mice (Fig. 5E).

**Figure 6:**
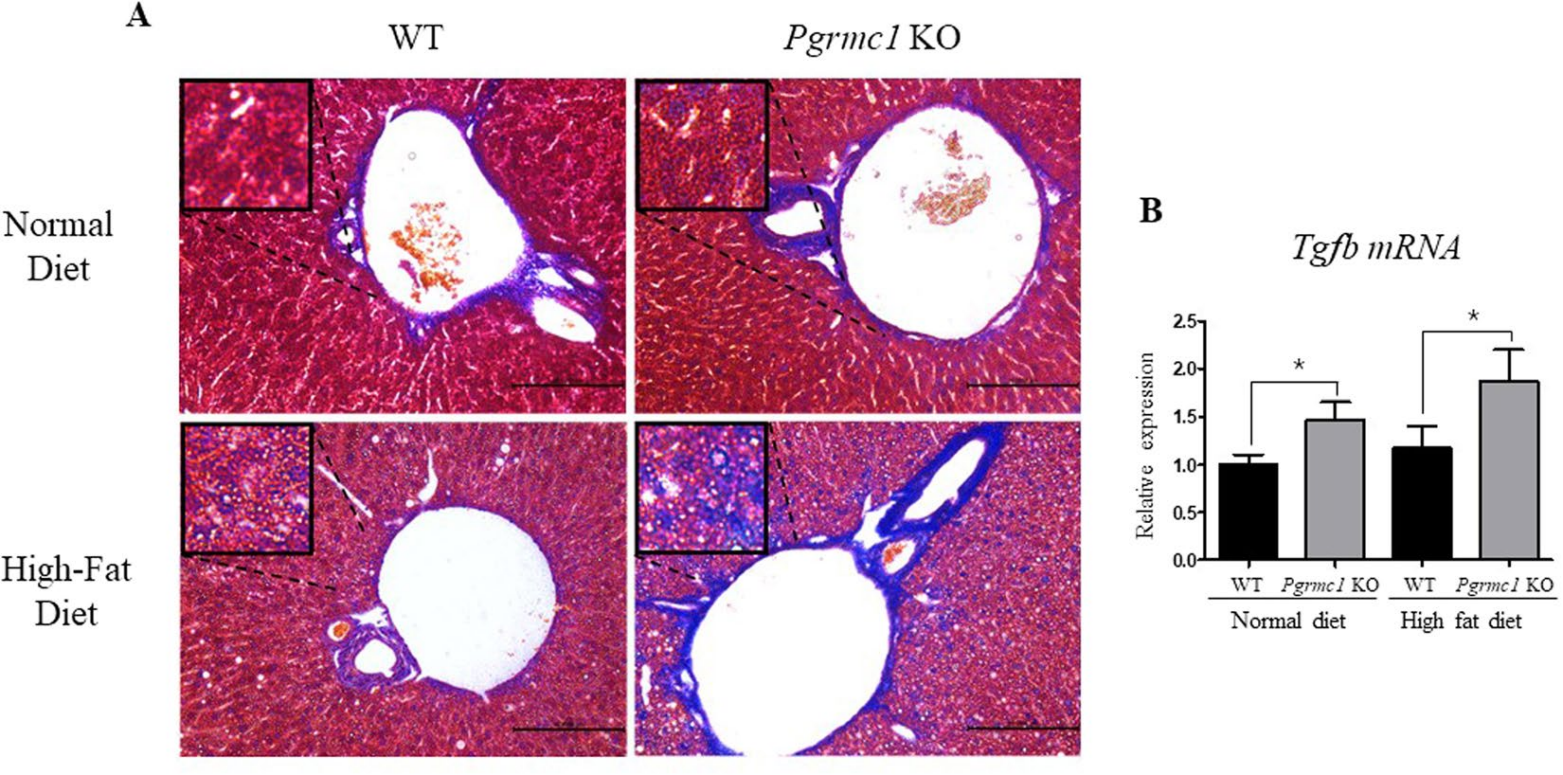
*Pgrmc1* KO mice possesses more susceptible condition for NASH with fibrosis. (**A**) Masson’s trichrome staining indicates fibroblasts produced by steatosis (Scale bar, 100 µm). (**B**) Increased mRNA level of fibrosis marker, *Tgf-b*, in livers of *Pgrmc1* KO mice. *Rplp0* mRNA was used as an internal control in real time PCR. Values represent means ± SD of at least 3 experiments. *P < 0.05. Number of mice used in experiment is at least 3 for each group.

To further evaluate NASH under condition of high-fat diets, we monitored the hepatic fibrosis using Masson’s trichrome staining. As expected, high-fat-fed *Pgrmc1* KO mice showed significantly higher ratio of blue-stained fibroblasts compared to those within WT mice fed with a normal diet and high fat diet, and the fibroblasts was mainly confined around the central vein of lobules (Fig. 6A). As another fibrosis marker, the level of *Tgf-β* mRNA also showed significant (*P* < 0.05) increase in both of normal diet-fed (1.46 fold) and high-fat-fed (1.58 fold) *Pgrmc1* KO mice than those of WT mice (Fig. 6B). These results suggest that *Pgrmc1* KO mice are genetically more prone to NAFLD-induced liver inflammation or NASH.

### Liver-specific regulation of PGRMC1 controls level of SREBP-1 and steatosis

**Figure 7:**
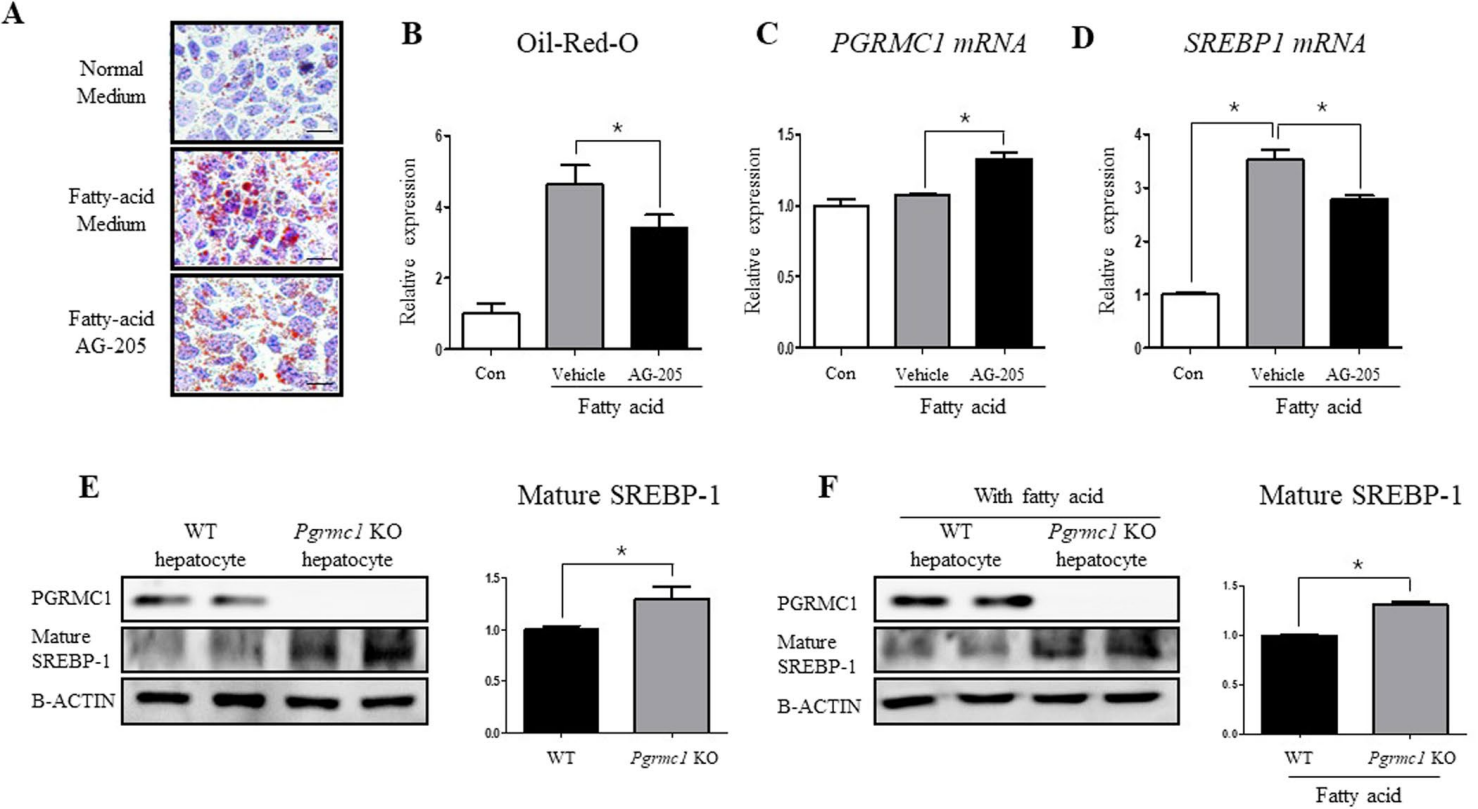
Liver-specific regulation of *Pgrmc1* controls hepatic fat accumulation through SREBP-1. (**A**,**B**) Palmitic acid (330 µM) and oleic acid (660 µM) were incubated for 48 hrs with high glucose medium. Fatty acid substantially increased Oil Red O stained area and AG-205 ameliorated fat accumulation in hepatocyte (Scale bar, 10 µm). (**C**,**D**) Fatty acids were incubated in same concentration as above for 4 hrs after pre-incubation of AG-205 for 12 hrs. *PGRMC1* mRNA level was increased after AG-205 treatment and *SREBP-1* mRNA level was inversely changed to *PGRMC1* mRNA level. (**E**,**F**) Cells were isolated from mice and maintained in high glucose medium for one day. Fatty acids (Palmitic acid; 330 µM, Oleic acid 660 µM) were treated for 24 hrs. Levels of mature SREBP-1 protein were increased in loss of *Pgrmc1*. *Rplp0* mRNA was used as an internal control in real time PCR. Beta actin was used as an internal control in western blot. Values represent means ± SD of at least 3 experiments. *P < 0.05.

For inducing lipogenesis, palmitic acid (330 μM) and oleic acid (660 μM) were treated for 48 hours and AG-205 was co-incubated in Hep3B cells. As a result, Oil-Red-O stained areas were significantly decreased (*P* < 0.05, 74.1%) in fatty acid + AG-205-treated group when compared to the fatty acid treated group, suggesting that lipid storage has been significantly reduced (Fig. 7A,B). Moreover, after treating fatty acids for 4 hours with pre-incubation of AG-205 for 12 hours, *PGRMC1* mRNA level increased (*P* < 0.05, 1.23 fold) in fatty acid + AG-205 group when compared to fatty acid group (Fig. 7C). On the one hand, *SREBP-1* mRNA level was inversely proportional to *PGRMC1* mRNA level, showing decreased level (*P* < 0.05, 75.8%) in the fatty acid + AG-205 group when compared to AG-205 group (Fig. 7D). These results show that up-regulation of *PGRMC1* down-regulates lipid accumulation in hepatocyte with decreased level of *SREBP-1*.

To investigate whether the lipogenic effect in the loss of *Pgrmc1* is liver-specific, we therefore introduced primary hepatocyte culture system and observed significantly increased (*P* < 0.05, 1.3 fold) level of mature SREBP-1 protein in *Pgrmc1* KO hepatocytes when compared to WT hepatocytes (Fig. 7E). Likewise, level of mature SREBP-1 protein also significantly increased (*P* < 0.05, 1.31 fold) in fatty acid treated *Pgrmc1* KO hepatocytes when compared to that of WT hepatocytes (Fig. 7F). The phenotypic characteristic of *Pgrmc1* was also confirmed by siRNA transfection with significantly increased (*P* < 0.05, 1.36 fold) level of mature SREBP-1 protein in *PGRMC1* knockdown Hep3B cell when compared to control cell (*P* < 0.05, 65%, Fig. S7).

## Discussion

The fatty liver is predisposed to injuries involving oxidant stress, which subsequently leads to inflammation^26^. NASH, the typical feature of NAFLD, occurs during accumulation of fatty acids following hepatic *de novo* lipogenesis or plasma lipid transport from extrahepatic tissues^27,28^. In addition, numerous studies reported that steatohepatitis increased the risk of HCC^28,29^. Interestingly, the present study shows that *Pgrmc1* KO mice are vulnerable to NAFLD triggered by a high-fat diet when compared to WT mice. Moreover, *Pgrmc1* KO mice were predisposed to liver inflammation and fibrosis; therefore, loss of *Pgrmc1* can increase the risk of hepatic diseases such as NAFLD and NASH under high-fat diets typical of western societies.

After mice were fed a high-fat diet, *Pgrmc1* KO mice showed significant increases in hepatic TG accumulation. *Pgrmc1* KO mice also showed significant increases in plasma FFA, while plasma TG revealed a corresponding reduction in levels. In a recent study, patients with NAFLD had accumulation of TG within the liver mainly from hepatic *de novo* lipogenesis rather than from plasma fatty acids^30^. Thus, we focused on hepatic lipogenesis to investigate the mechanism of increased hepatic steatosis in the loss of *Pgrmc1*.

To assess the transcription regulating gene involved in fatty acid synthesis of lipogenesis, we observed the increase of *Srebp-1* level following the loss of *Pgrmc1*. When SREBP-1 is the primary regulator of fatty acid synthesis and TG storage within the liver^31^, it was found to be inactive in its precursor form under non-high fat conditions. After being cleaved from the SCAP/SREBP-1 complex through signaling, SREBP-1 becomes mature and plays a role as a transcriptional factor for triggering hepatic *de novo* lipogenesis^32^. The signal for cleavage of the SCAP/SREBP-1 complex is transmitted from the PGRMC1/INSIG-1 complex, implying that PGRMC1- is closely involved with SREBP-1 function. More recently, it was reported that SCAP/SREBP-1 expression increases under a condition of low PGRMC1/INSIG-1 expression^25^. Likewise, the present study shows significantly increased levels of *Srebp-1* mRNA and mature SREBP-1 protein in the absence of *Pgrmc1*. Since induction of SREBP-1 has been linked to the accumulation of TG within the liver through *de novo* lipogenesis^33^, our data support the claim that hepatic TG accumulates within the *Pgrmc1* KO liver following an increase of *Srebp-1* levels.

In addition, our results provide evidence that *Pgrmc1* has a novel function as an inhibitory factor of hepatic *de novo* lipogenesis. As a *Srebp-1*-dependent protein, ACC plays a crucial role in lipogenesis and fatty acid synthesis^34,35^ as its protein product is responsible for the carboxylation of acetyl-CoA to malonyl-CoA. Furthermore, reduction of its inactive form was also used as an indicator of lipogenesis^36^. In this study, we observed an increased level of *Acc* in the absence of *Pgrmc1*, with increased SREBP-1 level. Moreover, we observed increased transcript level of fatty acid esterification enzymes, *Agpat* and *Mogat*, which may promote the growth of lipid droplets and TG accumulation in the absence of *Pgrmc1*^37,38^. These data, therefore, suggest that *Pgrmc1* KO mice are predisposed to lipogenesis in the liver. On the other hand, an increased level of fatty acid metabolism genes was observed after the loss of *Pgrmc1* (Figs S5 and S6), suggesting that TG accumulation in *Pgrmc1* KO liver was not induced by impaired fatty acid degradation.

Next, we observed an unexpected reduction in transcripts related to lipogenesis (*Scd1* mRNA) in the absence of *Pgrmc1* in both groups fed a normal diet and a high fat diet. *Pgrmc1* KO mice have a phenotype showing low levels of *Scd1* transcript, *Scd1* being the primary regulator in fatty acid synthesis, converting saturated fatty acids to monounsaturated fatty acids and preventing lipotoxicity^37–39^. As the *Scd1* gene plays a key role in preventing steatohepatitis, *Pgrmc1* deficiency could lead to a NASH-susceptible state, like that of *Scd1* KO mice^38^. Therefore, our data suggest that *Pgrmc1* KO mice are genetically more vulnerable to lipotoxicity acquired by hepatic steatosis. Meanwhile, plasma alanine aminotransferase (ALT) is a well-known marker for inflammation as it is located inside the liver cell and increases after hepatocyte damage. Lipotoxicity due to low *Scd1* levels after the loss of *Pgrmc1* should be linked to plasma ALT levels, and *Pgrmc1* KO mice showed enhanced levels of ALT over WT mice both in a normal diet and a high-fat diet. Though NASH is not always concomitant with ALT^40^, we supported our claim with the increased level of obesity-linked pro-inflammatory cytokines, *IL-6* and *TNF*^41^. These cytokines are well known mediators for inflammation or HCC induced by hepatic steatosis, because *IL-6* is involved in low-grade chronic inflammation and poor prognosis of NAFLD^42,43^. In addition, we observed an increase of macrophage infiltration in the absence of *Pgrmc1*, suggesting a high possibility for inflammation triggered by NASH. Likewise, considerable increases of fibroblasts and *Tgfb* transcript level also suggest susceptibility to fibrosis induced by steatosis in the loss of *Pgrmc1*^44,45^. Therefore, based on our results, *Pgrmc1* KO mice might have high risk factors for NASH including liver inflammation and fibrosis, and HCC triggered by a high-fat diet.

To gain insight into the correlation between Pgrmc1 and hepatic lipogenesis, we introduced AG-205 into Hep3B cells in a medium supplemented with fatty acids. Similar to the *in vivo* data, when *PGRMC1* mRNA level increased significantly after treatment with AG-205, fat storage significantly decreased in the *PGRMC1*-induced condition. In addition, *PGRMC1* directly regulated the mRNA level of *SREBP-1*, suggesting that lipogenesis could also be controlled by following *PGRMC1* promotion. Meanwhile, we further confirmed that lipogenic control of *Pgrmc1* is liver-specific by introducing a primary hepatocyte culture, observing increased levels of mature SREBP-1 in *Pgrmc1* KO hepatocytes. Likewise, we also decreased the level of PGRMC1 and observed an increased level of mature SREBP-1 in siRNA transfection.

In summary, our study suggests that the loss of *Pgrmc1* within the liver not only increases cellular lipid levels, but also enhances the risk for steatohepatitis. These findings are particularly interesting for cancer cell metabolism in light of reports that regard *Pgrmc1* as a clinical parameter for hepatocellular carcinoma^46^ and find that *Pgrmc1* is strongly expressed in different kinds of cancer^47,48^. We also suggest that the loss of *Pgrmc1* might enhance sensitivity for lipotoxicity in animals and possibly humans even under a low-lipid diet.

## Materials and Methods

### Antibodies

Rabbit polyclonal antibody for Beta-actin (sc-130656) was purchased from Santa Cruz biotechnology (Santacruz, CA, USA). Rabbit monoclonal antibody specific for Pgrmc1 (CST, #13856) was purchased from CST. Mouse monoclonal antibody specific for Srebp1 (sc-13551) was purchased from Santacruz.

### Animals

*Pgrmc1* KO mice were generated by TALEN method. Pgrmc1 specific TALEN plasmids were obtained from ToolGen, Inc. (South Korea). General methods for TALEN mediated KO mice production were previously described^49^. Briefly, Single dose of *Pgrmc1 TALEN mRNA* (50 ng/ul) was injected into the cytoplasm of C57BL/6 N mouse eggs with well recognized pronuclei and transferred into the oviducts of pseudo-pregnant foster mothers. To evaluate non-specific effects of TALEN, potential off-target sites were predicted as previously described^50^ (Table 1), and T7E1 assays were performed using genomic DNA samples from F0 mutant mice. For routine PCR genotyping of F1 progeny from selected F0 founder mice, the following primer pair was designed to amplify a 128 bp PCR product from wild-type mice and TALEN-induced mutant alleles: 5′-GGCTGCTGCACGAGATTTTC-3′ and 5′-GGTGGTTCGTCGTCGTCGTTG-3′ (Fig. 1).

C57BL/6N WT and *Pgrmc1* KO female mice were housed in the pathogen-free facility at Chungnam National University and fed with standard chow and high fat diet with water provided ad libitum. All mouse experiments were approved and performed in accordance with the Chungnam National University Animal Care Committee (CNU-00606). Sacrifice was performed by CO_2_ asphyxiation. Number of mice used for experiment was 3 for each normal diet groups and 4 for each high fat diet groups.

### Plasma TG, FFA and ALT level

All serums were diluted by 1/5 fold with PBS. Plasma TG and ALT level were measured with FUJI DRI-CHEM SLIDE (TG-1650, ALT-3250) by DRI-CHEM4000 (Fuji Film). Plasma FFA was measured with commercial kit (BM-FFA100) obtained from Biomax (Seoul).

### Liver TG level

Lipid extraction from liver was done with Folch method. 0.2 g of liver tissue was homogenized with beads and 0.9% NaCl solution. After mixing with chloroform and methanol (1:2), samples were let stand for 30 minutes on room temperature. Additional mix with chloroform and distilled water, lower phase was separated after centrifuged on 3,000 rpm, 20 mins. Steps after homogenization were repeated for 3 times. Samples were filtrated with filter paper and the flow-through was heated in water bath. By this step, chloroform phase was evaporated. After complete dry in dry oven, it was dissolved in chloroform and 2-propanol. Samples were stored in room temperature. TG level was analyzed with TG measurement solution (AM157S-K, Asan-Set) in 550 nm spectrophotometer

### RNA Isolation, reverse transcription, and qRT-PCR

Total RNA extracts from mouse liver or Hep3B cells were prepared using the TRIzol® Reagent (Thermo Fisher Scientific, MA, USA). Reverse transcription was performed with 1.5 µg of total RNA and Reverse transcriptase kit (SG-cDNAS100, Smartgene, United Kingdom) following manufacturer’s protocol. Quantitative PCR (real-time PCR) was carried out using specific primers (Table 2), Excel Taq Q-PCR Master Mix (SG-SYBR-500, Smartgene) and Stratagene Mx3000p (Agilent) equipped with a 96-well optical reaction plate. Negative controls, containing water instead of sample cDNA, were used in each plate. All experiments were run in triplicate and mRNA values were calculated based on the cycle threshold and monitored for melting curve

### Western blotting

Total liver and hep3B cell samples were homogenized by protein lysis buffer [T-PER (#78510, Thermo)] containing proteinase inhibitor (PMSF). Homogenized samples were quantified by Bradford assay with PRO-Measure solution (Intron, #21011) and proceeded to protein electrophoresis (SDS-PAGE) after 5 min of boiling in 100 °C. Gels were blotted by wet transfer with Bio-Rad Power Pac in 350 mA. Membranes were blocked for 1 hour in Skim milk and incubated with a primary antibody (see above) for overnight, 4 °C. After 3 times of washing with PBS-T or TBS-T, membranes were incubated with secondary antibodies (AE-1475 goat anti-rabbit, BS-0296G-HRP goat anti-mouse, Bioss) for 4 hours diluted with 1:5000 in skim milk or BSA. Results were detected with ECL solution (XLS025-0000, Cyanagen) and Chemi Doc (Fusion Solo, Vilber Lourmat).

### Histological analyses

For hematoxylin & eosin (H&E) staining, paraffin embedded tissue were cut by 4 μm and attached to silane coated slide. With serial hydration step in xylene, ethanol and distilled water, hematoxylin and eosin were stained. Region of interest was observed by light microscope. For Masson’s trichrome staining, slides were proceeded by manufacturer’s protocol (BioGnost, MST-100T).

For Oil-Red-O, frozen tissue were cut by 7 μM after embedded with OCT compound and attached to silane coated slide. After drying 10 mins in RT, slides were fixed with formalin for 20 mins and washed with running tap water for 10 mins. Slides were proceeded to rinse step with 60% isopropanol and stained with Oil Red O working solution (3 g/l) for 15 mins. After washing with 60% isopropanol, slides were stained with hematoxylin for 30 secs and rinsed with distilled water. Region of interest was observed by light microscope after mounted in aqueous mountant.

For immunofluorescence, frozen tissue were cut by 7 μM after embedded with OCT compound and attached to silane coated slide. After drying 10 mins in RT, slides were fixed with cold methanol for 20 mins in −20 °C. Slides were proceeded to permeabilization step with 0.5% triton x-100 and blocked by 3% BSA in TBS-T. Primary antibody (F4/80, ab6640, abcam) was incubated for O/N in 4 °C. After three times of wash with TBS-T, secondary antibody (A21202, Life Technologies) was incubated for 3 hrs in RT. Region of interest was observed in dark area with microscope (DMi8, Leica).

### Cell culture

Hep3B cells were cultured in DMEM-High glucose medium (LM 001-05). DMSO and AG-205(10 mg/ml, A1478, sigma) were treated for indicated hours before high fat treatment. Fatty acids (Palmitic acid 330 μM, Oleic acid 660 μM) were treated for each indicated hours.

SiRNA transfection was performed with lipofectamine 2000 (11668-027, Thermofisher) according to the manufacturer’s protocol. Negative control siRNA and PGRMC1 siRNA #1 and #2 were purchased from Bioneer (Daejoen, Korea). The sense sequences of PGRMC1 siRNA #1 and #2 were 5′-CAGUACAGUCGCUAGUCAA-3′ and 5′-CAGUUCACUUUCAAGUAUCA-U-3′.

Primary hepatocytes were isolated from mice by collagenase digestion and Percoll Gradient method. Briefly, mice were anesthetized, and the peritoneal cavity was opened. Livers were perfused with Ca^2+^ and Mg^2+^ free-HBSS containing EDTA (1 mM) (LB203-56, Welgene) and then digested with a collagenase solution containing liberase (Research grade, Sigma). Digested livers were removed and rinsed twice with HBSS and then gently teased with forceps until they were in solution. The cell suspensions were filtered through a sterile 40-μm nylon cell strainer (SPL) to remove undigested tissue and connective tissue. The cells were centrifuged for 5 min at 1000 rpm and resuspended with medium. The pellet suspensions were centrifuged using 35% Percoll for 15 min at 2000 rpm with the brake option off. After centrifugation, the healthy hepatocytes were pelleted as damaged hepatocytes, or nonparenchymal cells could not penetrate into 35% Percoll solution. The pellets were washed twice with DMEM supplemented with 5%FBS, and then seeded into six well tissue culture plates. After 24 hrs, nonadherent cells were removed by aspiration.

### Statistical analysis

Data are reported as mean ± SD. Differences between means were obtained by Student’s t-test was performed using Graph Pad Software (GraphPad Inc., San Diego, CA).

## Electronic supplementary material

Supplementary material
