# Supplementary material for: Loss of progesterone receptor membrane component 1 promotes hepatic steatosis via the induced de novo lipogenesis
Source: Sci Rep. 2018 Oct 24;8:15711. doi: 10.1038/s41598-018-34148-6 (PMC6200820; doi:10.1038/s41598-018-34148-6)
Supplement: Supplementary file 1 — Supplementary material [file 41598_2018_34148_MOESM1_ESM.pdf]

**Supplementary Information**

# Loss of progesterone receptor membrane component 1 promotes hepatic steatosis via the induced *de novo* lipogenesis

**Sang R. Lee[1], Sun Woo Kwon[1], Pelin Kaya[1], Young Ho Lee[1], Jong Geol Lee[2], Globinna Kim[2], In-Jeoung Baek[2*], and Eui-Ju Hong[1*]**

[1]Laboratory of Biochemistry, College of Veterinary Medicine, Chungnam National University, Daejeon 34134, Republic of Korea

[2]Department of Convergence Medicine, University of Ulsan College of Medicine, Asan Medical Center, Seoul 05505, Republic of Korea.

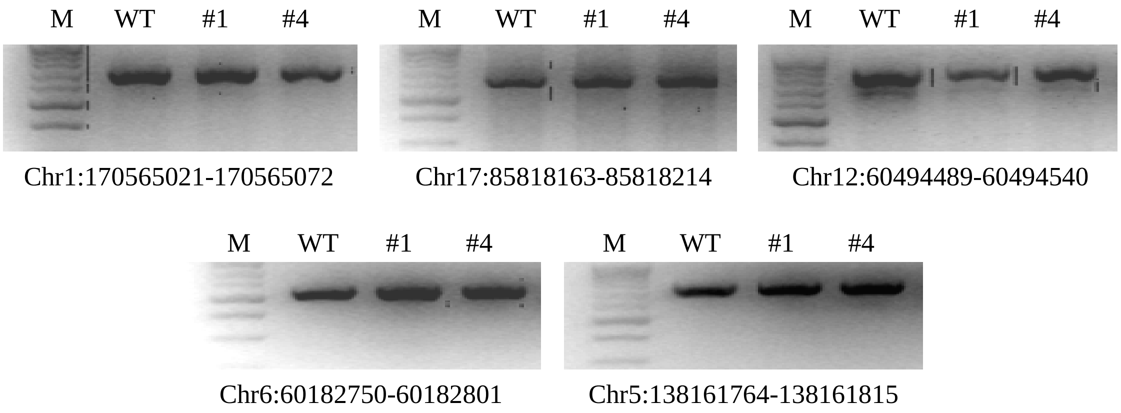


Fig. S1. Analysis of off-target effects of TALEN targeting the *Pgrmc1* gene. T7E1 assays examining the putative off-target effects of *Pgrmc1*-TALEN. The integrity of potential off-target sites as listed in Table 1 was examined in founder mice.

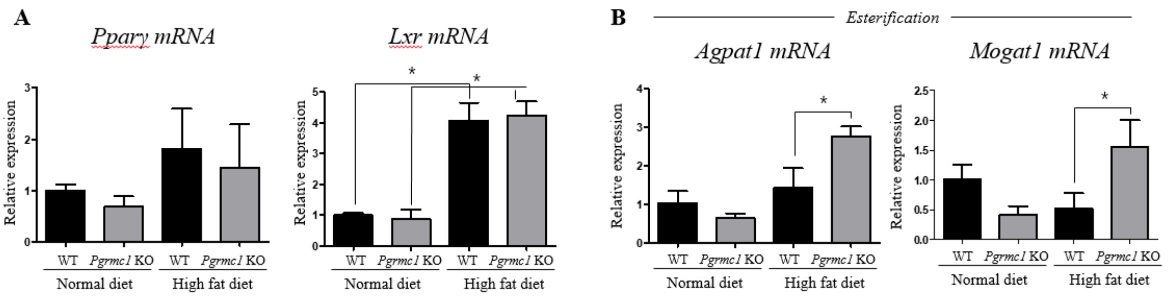


Fig. S2. Transcriptional regulators and TG esterification enzymes related to *Srebp1* gene. Major fatty acid synthesis transcriptional regulator genes which are on the crosstalk with SREBP-1 (A). Genes regulates TG esterification in liver (B). After fed a high fat diet for 1 month, mRNA levels were determined by quantitative RT-PCR. *Rplp0* mRNA was used as an internal control. Values represent means ± SD at least 3 experiments. *, P<0.05. Number of mice used in experiment for normal diet group is at least 3 and for high fat diet groups is at least 4 for each group.

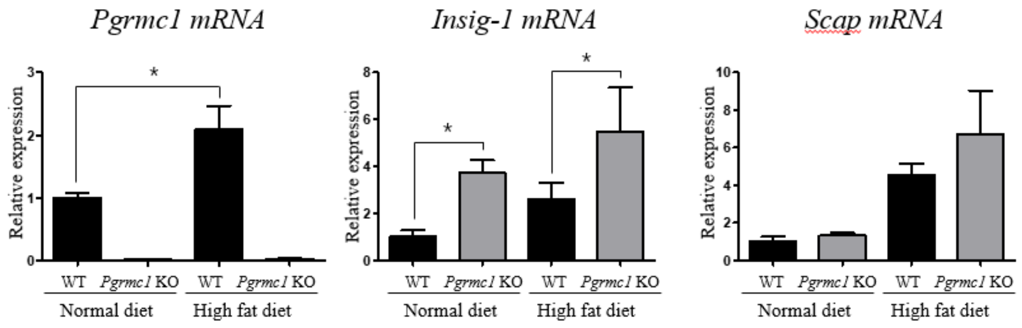


Fig. S3. Structural components related to *Srebp-1* gene. Genes forming complex with *Srebp-1* in endoplasmic reticulum. After fed a high fat diet for 1 month, mRNA levels were determined by quantitative RT-PCR. *Rplp0* mRNA was used as an internal control. Values represent means ± SD at least 3 experiments. *, P<0.05. Number of mice used in experiment for normal diet group is at least 3 and for high fat diet groups is at least 4 for each group.

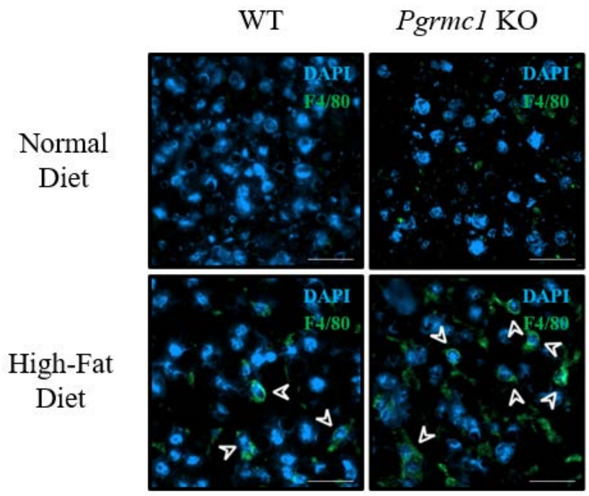


Fig. S4. Immunofluorescence staining of mouse macrophage marker, F4/80. Cytoplasm stained with green represents positive for F4/80. Nucleus stained with blue representing DAPI was used as an internal control. Number of mice used in experiment for normal diet group is at least 3 and for high fat diet groups is at least 4 for each group.

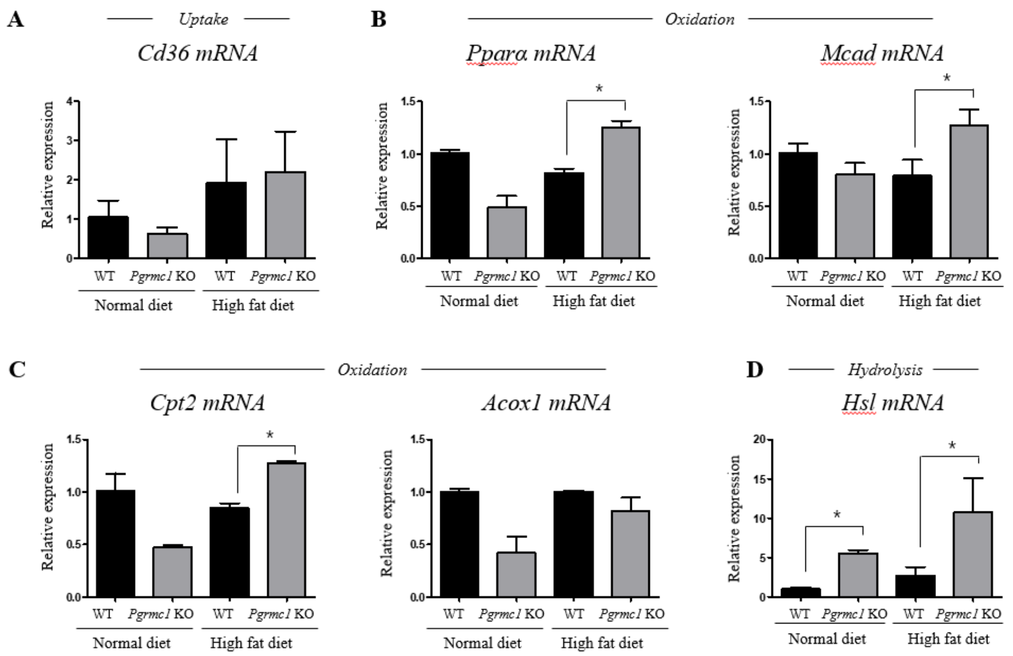


Fig. S5. Levels of fatty acid metabolic genes increased with TG accumulation. Genes related to fatty acid uptake, oxidation, and hydrolysis (A - D). After fed a high fat diet for 1 month, mRNA levels were determined by quantitative RT-PCR. *Rplp0* mRNA was used as an internal control. Values represent means ± SD at least 3 experiments. *, P<0.05. Number of mice used in experiment for normal diet group is at least 3 and for high fat diet groups is at least 4 for each group.

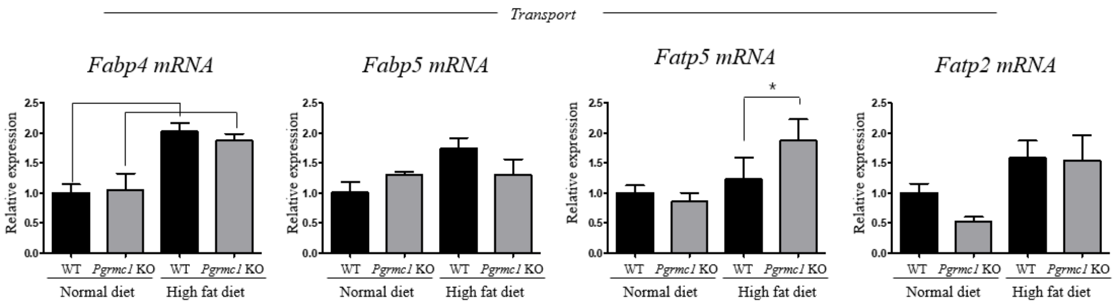


Fig. S6. Levels of fatty acid transport related genes. After fed a high fat diet for 1 month, mRNA levels were determined by quantitative RT-PCR. *Rplp0* mRNA was used as an internal control. Values represent means ± SD at least 3 experiments. *, P<0.05. Number of mice used in experiment for normal diet group is at least 3 and for high fat diet groups is at least 4 for each group.

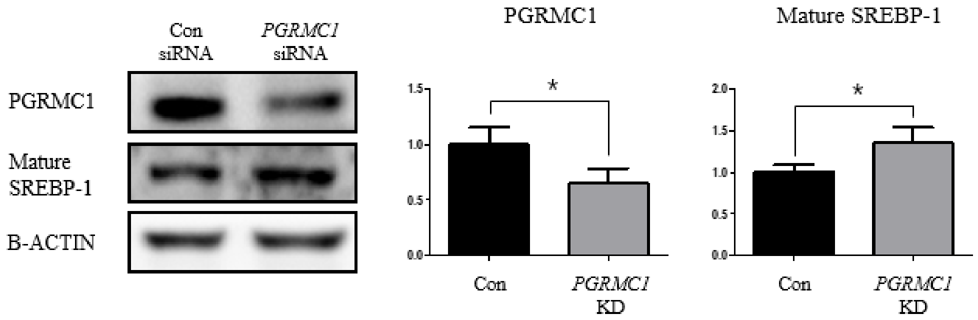


Fig. S7. Level of mature SREBP-1 protein in *PGRMC1* siRNA transfection. After transfected with siRNA, cells were treated with fatty acids (Palmitic acid; 330μM, Oleic acid; 660μM) for 24 hrs. Beta actin was used as an internal control. Values represent means ± SD at least 3 experiments. *, P<0.05. All experiments were repeated at least 3 times.

Fig. 1B

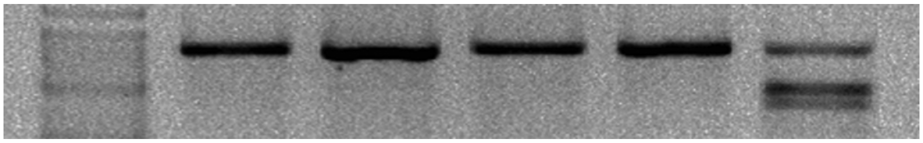

Fig. 1C

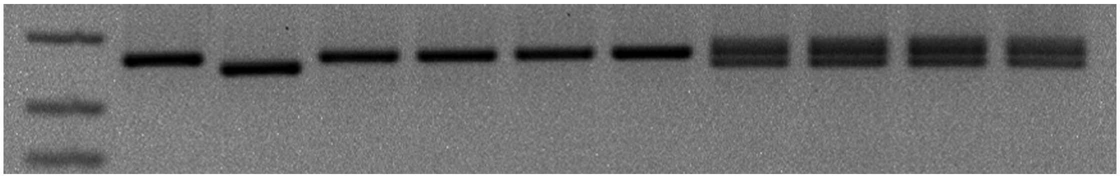

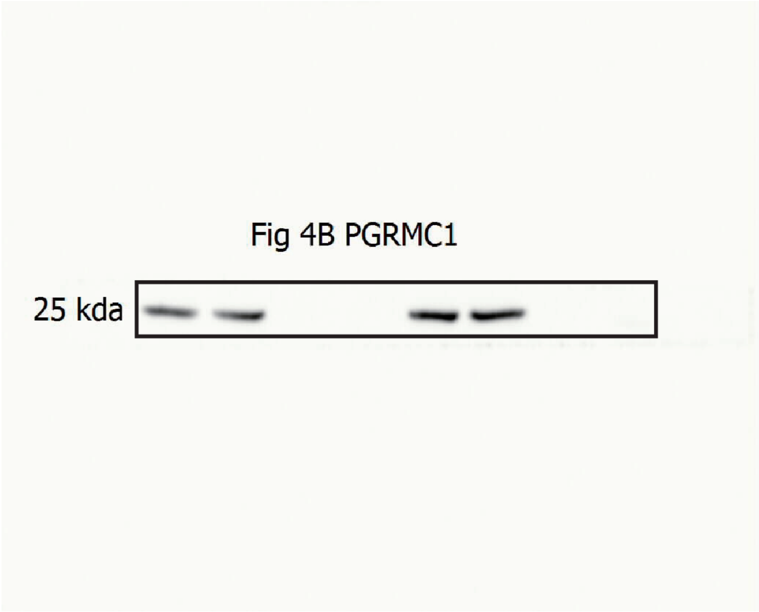
Fig 4B PGRMC1
25 kda

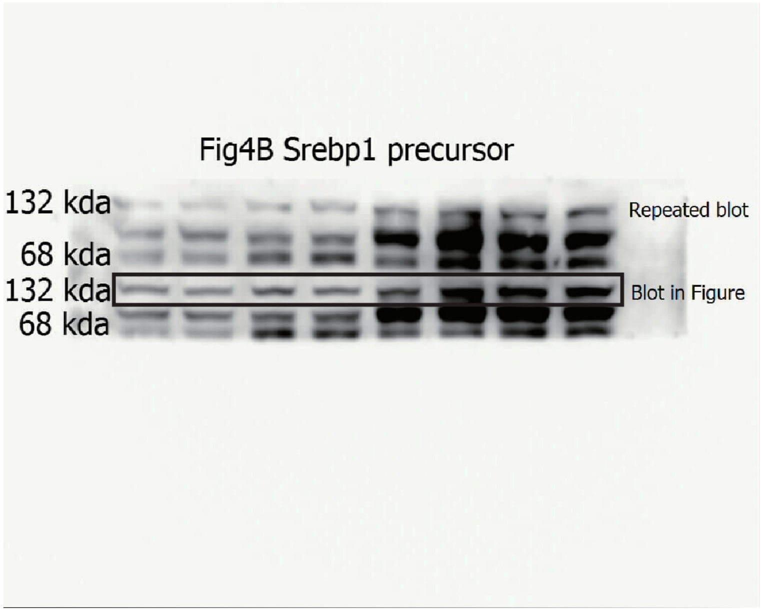
Fig4B Srebp1 precursor
132 kda
68 kda
132 kda
68 kda
Repeated blot
Blot in Figure

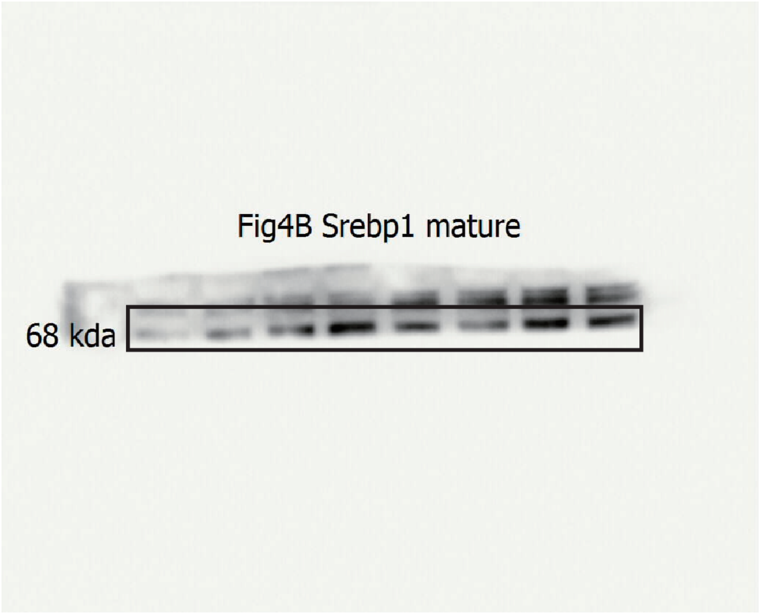
Fig4B Srebp1 mature
68 kda

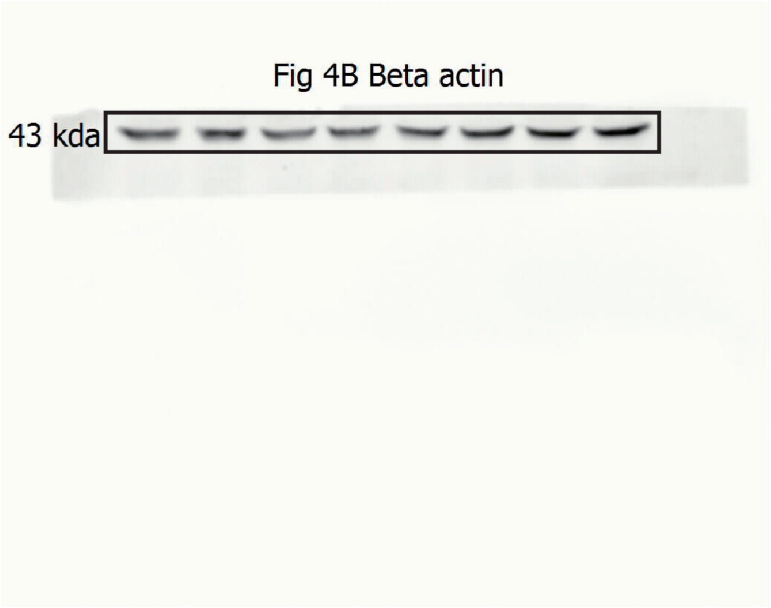
Fig 4B Beta actin
43 kda

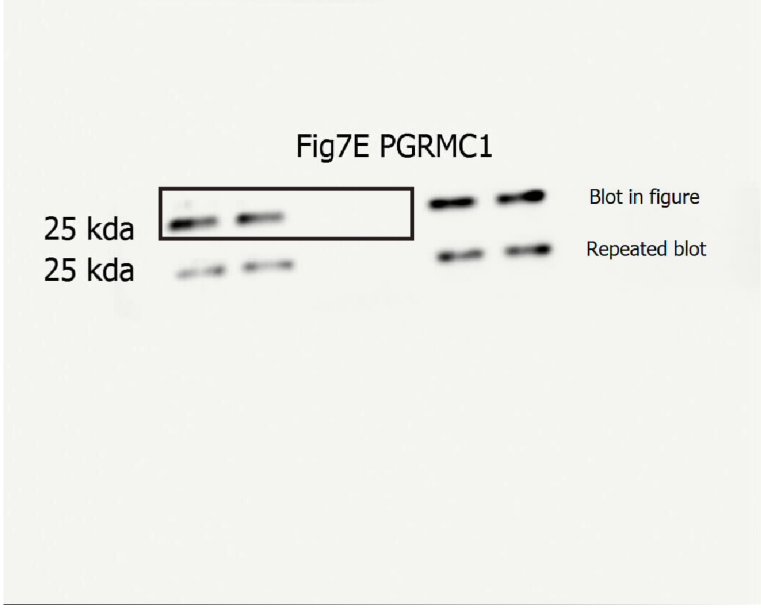
Fig7E PGRMC1
25 kda
25 kda
Blot in figure
Repeated blot

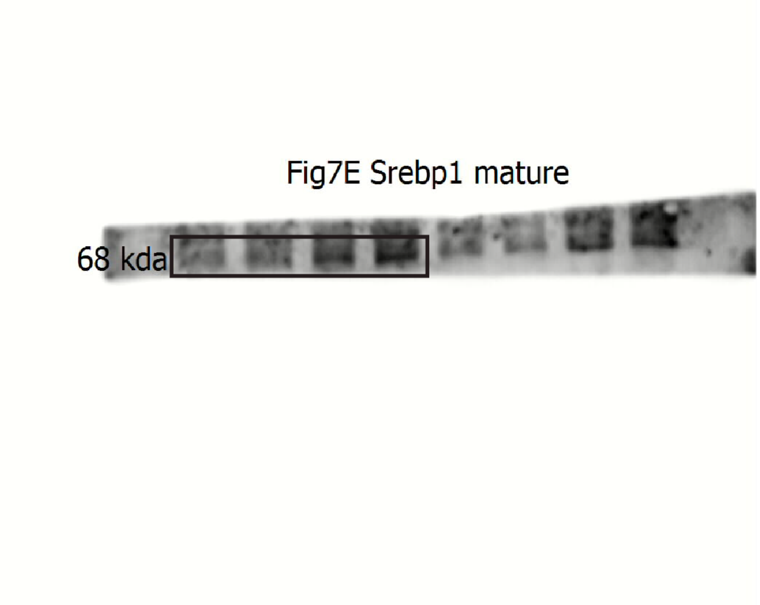
Fig7E Srebp1 mature
68 kda

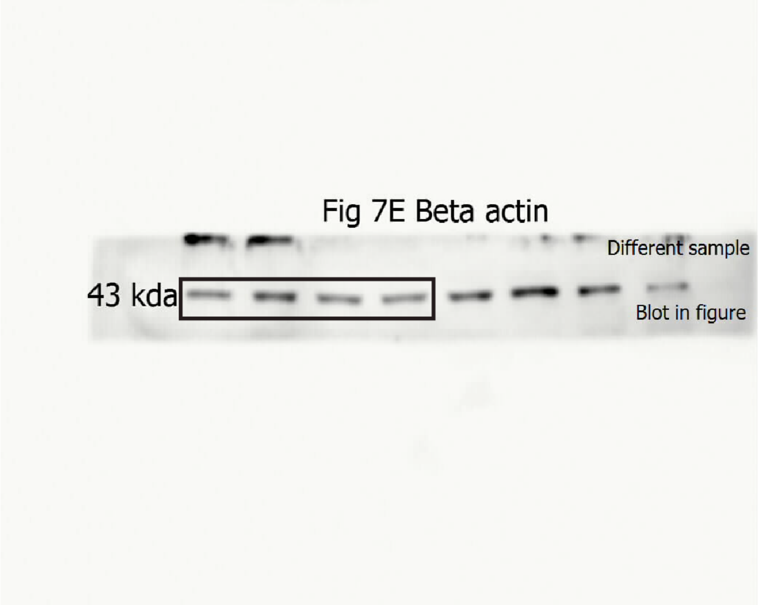
Fig 7E Beta actin
Different sample
43 kda
Blot in figure

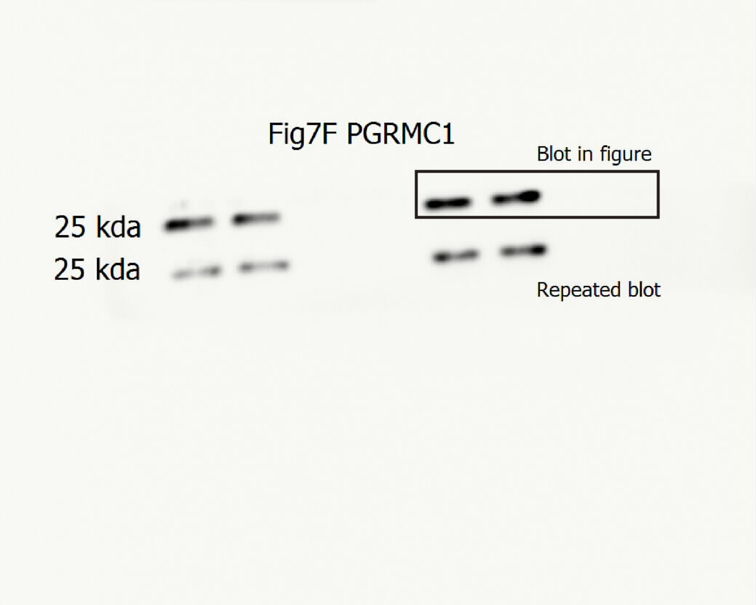
Fig7F PGRMC1
Blot in figure
25 kda
25 kda
Repeated blot

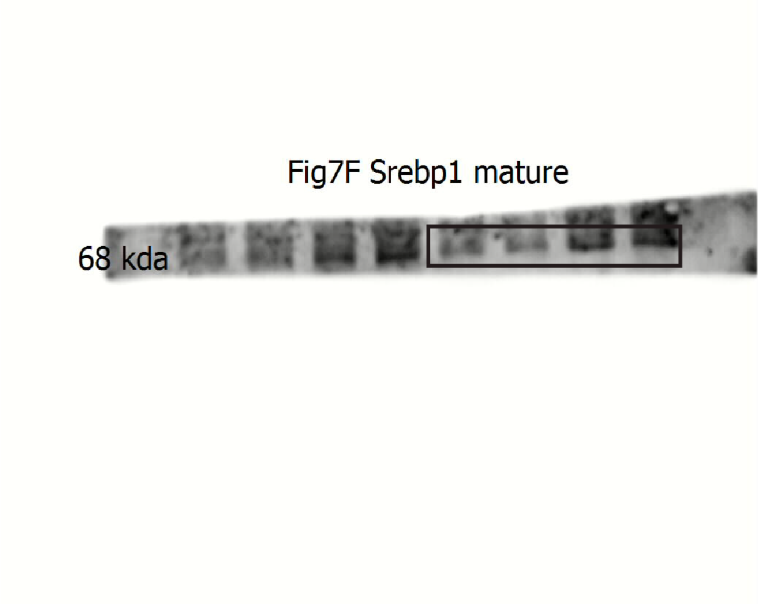
Fig7F Srebp1 mature
68 kda

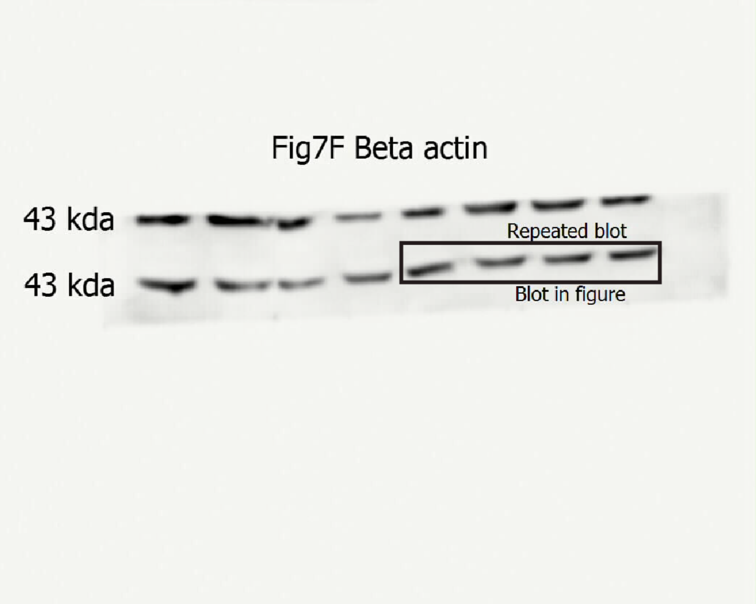
Fig7F Beta actin
43 kda
Repeated blot
43 kda
Blot in figure

Fig S1

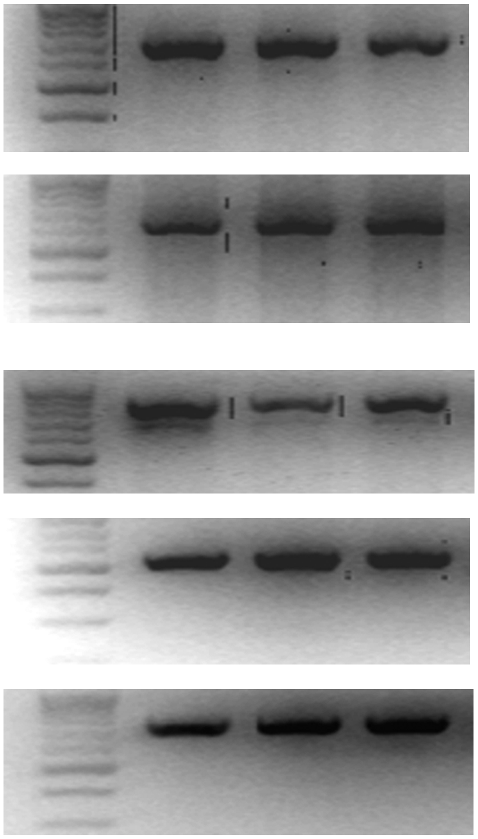

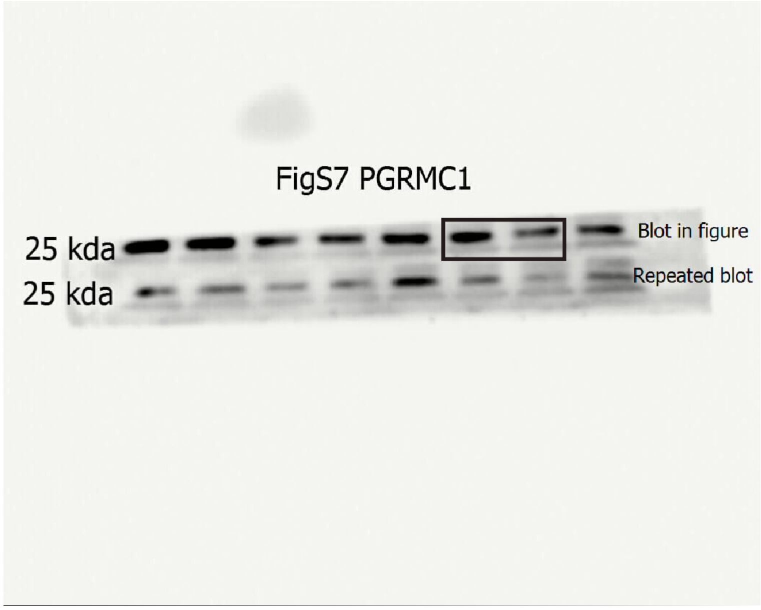
FigS7 PGRMC1
25 kda
25 kda
Blot in figure
Repeated blot

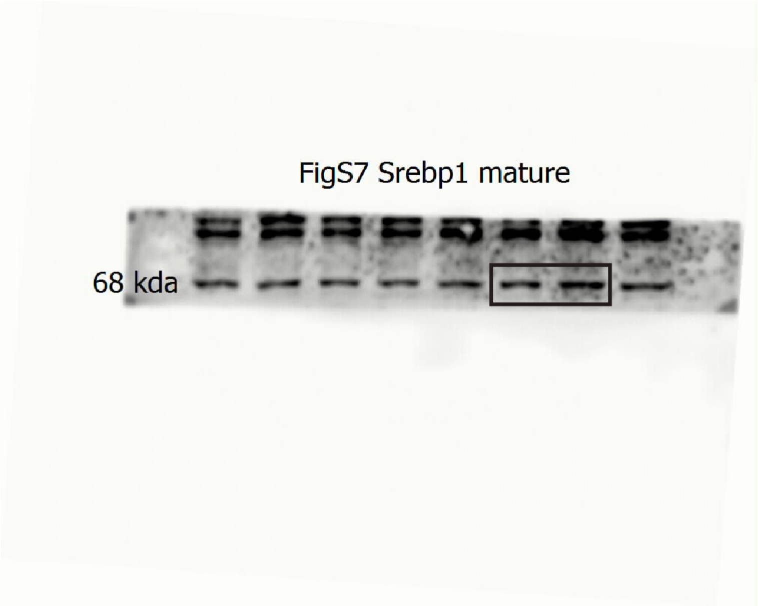
FigS7 Srebp1 mature
68 kda

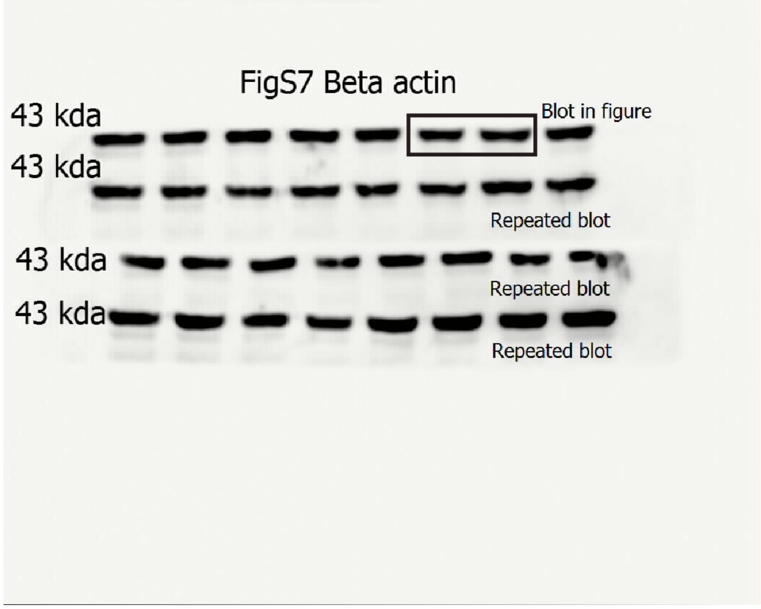
FigS7 Beta actin
43 kda
43 kda
Blot in figure
Repeated blot
43 kda
Repeated blot
43 kda
Repeated blot
